# Synthesis, Structure and Cytotoxic Activity of New Acetylenic Derivatives of Betulin

**DOI:** 10.3390/molecules18044526

**Published:** 2013-04-17

**Authors:** Stanisław Boryczka, Ewa Bębenek, Joanna Wietrzyk, Katarzyna Kempińska, Maria Jastrzębska, Joachim Kusz, Maria Nowak

**Affiliations:** 1 Department of Organic Chemistry, Medical University of Silesia, 4 Jagiellonska Str., 41-200 Sosnowiec, Poland; 2 Department of Experimental Oncology, Ludwik Hirszfeld Institute of Immunology and Experimental Therapy, Polish Academy of Sciences, 12 R. Weigla Str., 53-114 Wrocław, Poland; 3 Department of Solid State Physics, Institute of Physics, University of Silesia, 4 Uniwersytecka Str., 40-007 Katowice, Poland; 4 Department of Physics of Crystals, Institute of Physics, University of Silesia, 4 Uniwersytecka Str., 40-007 Katowice, Poland

**Keywords:** acetylenic betulins, synthesis, crystal structure, cytotoxic activity

## Abstract

A new series of betulin derivatives containing one or two pharmacophores bearing an acetylenic and carbonyl function at the C-3 and/or C-28 positions has been synthesized and characterized by ^1^H- and ^13^C-NMR, IR, MS and elemental analyses. The crystal structure of 28-*O*-propynoylbetulin was determined by X-ray structural analysis. All new compounds, as well as betulin, were tested *in vitro* for their antiproliferative activity against human SW707 colorectal, CCRF/CEM leukemia, T47D breast cancer, and against murine P388 leukemia and Balb3T3 normal fibroblasts cell lines. Most of the compounds showed better cytotoxicity than betulin and cisplatin used as reference agent. 28-*O*-Propynoylbetulin was the most potent derivative, being over 500 times more potent than betulin and about 100 times more cytotoxic than cisplatin against the human leukemia (CCRF/CEM) cell line, with an ID_50_ value of 0.02 μg/mL.

## 1. Introduction

Betulin [lup-20(29)-ene-3β,28-diol, C_30_H_50_O_2_, **1**] also known as betulinic alcohol, is a pentacyclic triterpene of the lupane type which was one of the first natural products identified and isolated from plants as a pure chemical substance in 1788 by Lowitz [1]. The still growing interest in betulin (**1**) and its derivatives results from their wide spectrum of biological activities such as anticancer, antiviral, anti-inflammatory, antibacterial and hepatoprotective properties [2,3,4]. Despite the fact that betulin (**1**) has been known for over 200 years, the X-ray crystal structure of this compound was investigated for the first time in 2010 by Drebushchak and in 2011 by Boryczka as betulin-EtOH and betulin-DMSO solvates, respectively [5,6]. Betulin (**1**) has three available sites for simple chemical modification, namely the secondary hydroxyl group at position C-3, the primary hydroxyl group at position C-28 and the isopropenyl side chain at position C-19.

**Figure 1:**
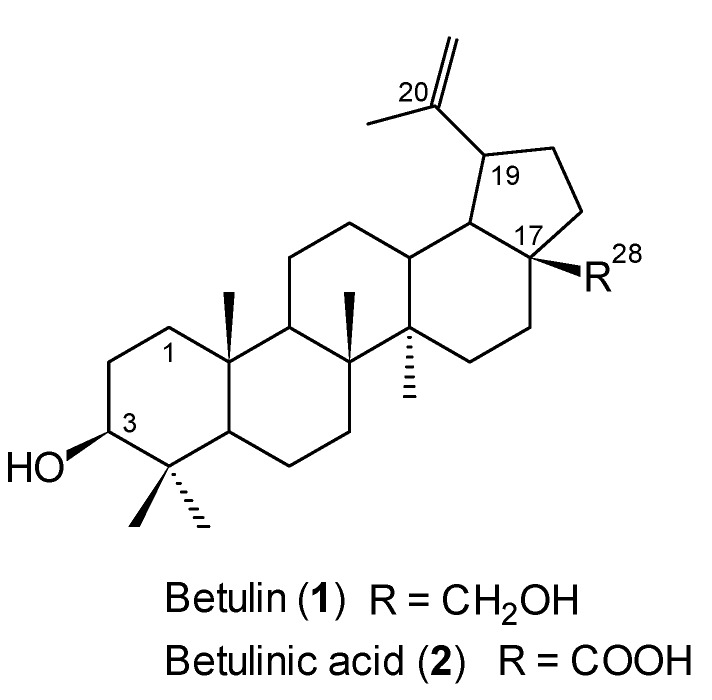
Structure of betulin (**1**) and betulinic acid (**2**).

Compound **1** can be easily converted in high yield to betulinic acid (**2**), which possesses a wide spectrum of biological and pharmacological activities and is now a very attractive and promising agent for clinical treatment for various types of cancer [7,8,9]. The chemical structures of these two compounds differ only in one group at C-17 [betulin (**1**) R=CH_2_OH, betulinic acid (**2**) R=COOH], and they are shown in Figure 1. In contrast to **2**, betulin (**1**) has been described as inactive or less active against cancer cells [3,10]. However, the results of recent reports suggest that betulin (**1**) shows a significant cytotoxic activity in a similar way as betulinic acid (**2**) [11,12].

The high content of betulin (**1**) in white birch bark (up to 30%) compared to betulinic acid (**2**) (up to 2.5%), make it an important starting material for synthesis of new compounds with various interesting medical properties. In the last few years a large number of betulin derivatives have been reported to possess anticancer, anti-inflammatory, anti-HIV and anti-leishmanial activity [2,13,14,15,16].

It is well known that the carbon-carbon triple bond is one of the most important functional groups in medicinal and organic chemistry [17]. Nevertheless, only a few reports have been described the synthesis of acetylenic derivatives of betulin which possess useful biological activity such as cytotoxic and antiviral properties [18,19,20,21,22]. It is interesting to note that the presence of an alkynyl group offers a possibility for further functionalization of betulins [23,24].

As an extension of our study on the development of novel acetylenic derivatives with potential anticancer activity [25,26], we became interested in the synthesis and evaluation of cytotoxicity of new derivatives of betulin containing one or two pharmacophores including both carbonyl group and a triple bond at the C-3 and/or C-28 positions. In order to investigate structure-activity relationships for the anticancer effects, two types of reagents were used for modifications of the hydroxyl groups of betulin (**1**), namely acetylene chloroformate and propynoic acids. All new synthesized compounds were tested for cytotoxic activity against human: breast cancer (T47D), leukemia (CCRF/CEM), colorectal adenocarcinoma (SW707), and murine: leukemia (P388) as well as normal fibroblasts (Balb3T3) cell lines in order to obtain more information about the influence of triple bond and carbonyl group on anticancer activity in this class of compounds. 

## 2. Results and Discussion

### 2.1. Chemistry

**Scheme 1:**
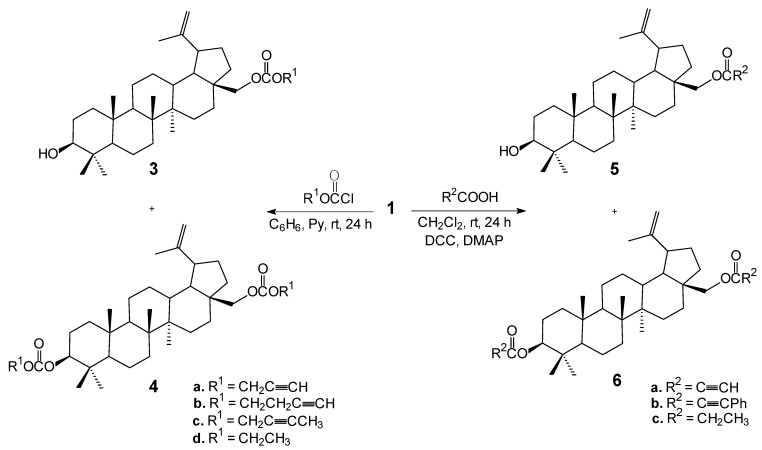
Synthesis of acetylenic derivatives of betulin **3**–**6**.

**Scheme 2:**
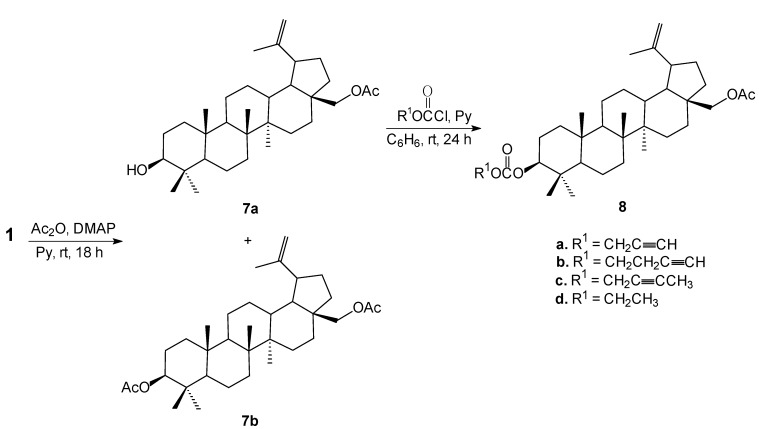
Synthesis of betulin derivatives **7** and **8**.

Synthesis of acetylenic derivatives of betulin **3**–**9** was accomplished starting with betulin (**1**). Compound **1** was isolated from birch bark by extraction with dichloromethane. The crude betulin (**1**) was purified by column chromatography using a mixture of chloroform and ethanol as an eluent. Full structural elucidation of the betulin (**1**) was performed using ^1^H- and ^13^C-NMR and IR spectroscopy, and the assignments were done based on our analysis and related literature [6,27]. The synthesis of acetylenic derivatives of betulin **3**–**9** is described in Scheme 1 and Scheme 2.

Treatment of betulin (**1**) with the corresponding chloroformate in benzene in the presence of pyridine at room temperature gave a mixture of mono- **3** and diesters **4**, in which the first isomers **3** were the major products due to the fact that reactivity of the hydroxyl group at C-28 is much higher than that of the C-3 one. The mixtures were separated by column chromatography to afford pure products **3** and **4** in 54%–69% and 23%–28% yields, respectively. The reaction of betulin (**1**) with propynoic acid or phenylpropynoic acid or propanoic acid in dichloromethane in the presence of catalytic amount of dicyclohexylcarbodiimide (DCC) and 4-dimetylaminopyridine (DMAP) afforded mixtures of mono- **5** and diester **6**, which were separated by column chromatography to give pure **5** and **6** in 50%–86% and 11%–27% yields, respectively. 

For the synthesis of compounds **8** (Scheme 2) the corresponding monoacetylated substrate **7a** was prepared, according to the method described in literature [28]. The synthesis of 28-*O*-acetylbetulin (**7a**) was achieved by selective acetylation of the primary hydroxyl group of betulin (**1**) with an equimolar amount of acetic anhydride in dichloromethane in the presence of DMAP and pyridine at room temperature. The reaction also gave small amount of 3,28-*O*,*O*'-diacetylbetulin (**7b**) which was separated by column chromatography.

The 28-*O*-acetylbetulin (**7a**) was then treated with corresponding chloroformates in benzene in the presence of pyridine under mild conditions to afford 28-*O*-acetyl-3-*O*'-alkynyloxycarbonylbetulins **8** in good yields (63%–89%). The structure of all new compounds **3**–**8** were determined on the basis of their ^1^H, ^13^C-NMR, IR and MS spectra, together with elemental analyses. In addition, crystal structure of **5a** was determined by X-ray structural analysis. For the first time, crystal structure of an acetylenic derivative of betulin-DMSO solvate is reported here.

### 2.2. Crystal Structure

**Figure 2:**
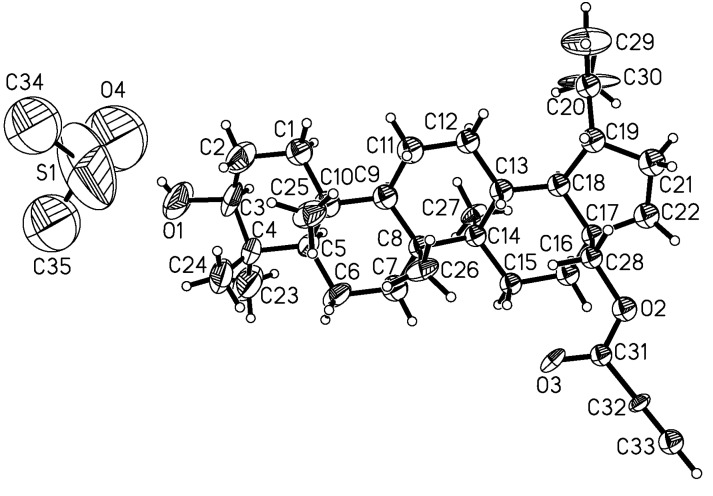
Molecular structure with atomic numbering scheme of 28-*O*-propynoylbetulin – DMSO solvate **5a** with displacement ellipsoids of 50% probability.

Crystals of 28-*O*-propynoylbetulin (**5a**) for X-ray structural analysis were grown from a DMSO solution as a 28-*O*-propynoylbetulin-DMSO solvate of 1:1 stoichiometry. The crystal structure of **5a** is shown in Figure 2. The compound crystallizes in the hexagonal system (space group P6_5_) with a = 2.6511 (1) nm, b = 2.6511 (1) nm, c = 8.3895 (2) nm, α = 90°, β = 90°, γ = 120°, V = 510.6 (1) nm^3^, Z = 6. The six-membered rings have a chair conformation, while the cyclopentane ring adopts a twisted conformation. All ring junctions in the 28-*O*-propynoylbetulin-DMSO solvate are *trans*-fused. A similar ring conformation was also observed in betulin-DMSO solvate [6], 3,28-*O*,*O*'-diacetylbetulin [29] and betulinic acid-DMSO solvate [30].

The methyl groups at carbon atoms C-24, C-25, C-26, C-27 occupy the axial positions, whereas the methyl group at C-23 and the isopropenyl group at C-19 are equatorial. The torsion angle C29-C20-C19-C21 describing the orientation of the isopropenyl group is equal to 105.4°. This conformation is very similar to that observed for the corresponding angles in 3,28-*O*,*O*'-diacetylbetulin (107.18°) [29] and in betulin-ethanol solvate (88.6°) [5] but differs from that observed for the structure of betulin-DMSO solvate (−96.8°) [6], and for betulinic acid-DMSO solvate (−112.2°) [30]. 

The OH group at C-3 is an equatorial orientation, while the substituted hydroxymethyl group is attached to the atom C-17 in an axial orientation. The oxygen atom O-4 of DMSO and the hydroxyl group at C-3 of betulin (**1**) are involved in the O1 – H1…O4 bond, which is relatively strong with short H…O distance (1.86 Å) and almost linear directionality (169.4°). 

### 2.3. Cytotoxic Activity

Cytotoxic activities of all newly synthesized compounds including betulin (**1**) were tested in SRB or MTT (in the case of leukemia cells) assay for their antiproliferative activity *in vitro* against three human cancer cell lines: SW707 (colorectal adenocarcinoma), CCRF/CEM (leukemia), T47D (breast cancer), and murine leukemia P388 as well as Balb3T3 normal fibroblasts cell lines. The results of the cytotoxic activity *in vitro* were expressed as an IC_50_ value (μg/mL), *i.e.*, the concentration of compound which inhibits the proliferation of 50% of tumor cells as compared to the control untreated cells. Cisplatin was used as a reference cytotoxic agent (positive test control). A value of less than 4 μg/mL was considered as an antiproliferative activity criterion for synthetic compounds [31]. The results of the cytotoxicity studies are summarized in Table 1.

**Table 1:** Cytotoxic activity of betulin (**1**), acetylenic betulins **3**–**8** and cisplatin reference compound against the cells of the tested human and murine cancer cell lines.

Compd.			Cytotoxic activity IC_50_ [µg/mL]				
			Human			Murine	
	**R^1^**	**R^2^**	**T47D**	**CCRF/CEM**	**SW707**	**P388**	**Balb3T3**
**1**	-	-	32.4 ± 10.7	10.9 ± 5.5	22.9 ± 15.4	5.5 ± 3.3	47.3 ± 7.9
**3a**	CH_2_C≡CH	-	7.2 ± 1.7	3.2 ± 0.4	9.2 ± 7.1	2.9 ± 0.2	3.9 ± 2.8
**3b**	CH_2_CH_2_C≡CH	-	6.9 ± 2.3	5.2 ± 1.4	22.6 ± 11.9	3.6 ± 1.3	19.5 ± 13.9
**3c**	CH_2_C≡CCH_3_	-	20.1 ± 5.3	6.8 ± 2.9	36.9 ± 6.3	5.5 ± 2.8	28.4 ± 17.6
**3d**	CH_2_CH_3_	-	6.6 ± 1.3	4.1 ± 0.2	20.8 ± 14.7	3.8 ± 1.9	19.1 ± 13.5
**4a**	CH_2_C≡CH	-	Neg	Neg	Neg	33.4 ± 1.7	Neg
**4b**	CH_2_CH_2_C≡CH	-	Neg	Neg	Neg	Neg	Neg
**4c**	CH_2_C≡CCH_3_	-	Neg	Neg	Neg	50.4 ± 31.1	Neg
**4d**	CH_2_CH_3_	-	69.1 ± 12.4	52.6 ± 8.3	Neg	46.8 ± 21.6	Neg
**5a**	**-**	C≡CH	9.1 ± 1.9	0.02 ± 0.001	14.9 ± 3.3	0.4 ± 0.1	0.3 ± 0.05
**5b**	**-**	C≡CPh	Neg	49.0 ± 9.8	Neg	Neg	Neg
**5c**	**-**	CH_2_CH_3_	12.1 ± 4.4	8.1 ± 0.9	29.2 ± 24.4	3.3 ± 0.8	32.3 ± 23.0
**6a**	**-**	C≡CH	16.2 ± 4.3	9.9 ± 7.0	9.5 ± 1.9	31.9 ± 1.4	21.1 ± 2.6
**6b**	**-**	C≡CPh	Neg	66.0 ± 2.8	Neg	Neg	Neg
**6c**	-	CH_2_CH_3_	26.6 ± 6.8	Neg	42.3 ± 9.4	68.1 ± 14.8	Neg
**7a**	-	-	4.9 ± 0.6	3.4 ± 0.3	5.1 ± 2.5	12.5 ± 14.7	29.7 ± 35.8
**7b**	-	-	Neg	34.1 ± 6.4	Neg	40.2 ± 5.0	Neg
**8a**	CH_2_C≡CH	-	Neg	Neg	Neg	Neg	Neg
**8b**	CH_2_C≡CCH_3_	-	Neg	Neg	Neg	46.7 ± 16.2	Neg
**8c**	CH_2_CH_2_C≡CH	-	Neg	Neg	Neg	Neg	Neg
**8d**	CH_2_CH_3_	-	Neg	Neg	Neg	Neg	Neg
**Cisplatin**			3.1 ± 1.0	2.0 ± 0.5	2.2 ± 0.5	0.5 ± 0.3	2.7 ± 0.3

Neg – negative in the concentration used.

As shown in Table 1, betulin (**1**) exhibited notably lower anticancer activity against the investigated cancer cell lines, with IC_50_ values in the 10.9–47.3 μg/mL range, except for leukemia P388 cell line (IC_50_ = 5.5 μg/mL). In this case, the selectivity index (SI) value, expressed as the ratio of IC_50_ of normal fibroblast/ IC_50_ on corresponding cancer cell line, is equal to 8.6. The introduction of an acetylenic formate group at the C-28 led to an increase of activity against the tested human and murine cancer lines. In the series of derivatives **3**, the most active compound **3a** was three to four times more cytotoxic than betulin (**1**) against cancer cells applied. Moreover, the degree of selectivity of this compound was lower than that of betulin (**1**) and derivatives **3b**–**d**. By comparing the cytotoxic activities of monoesters **3a**–**c**, it was found that the cytotoxic potency may be dependent on the length of the alkynyl chain and position of the triple bond on the formate group at the C-28 of betulin derivatives **3**. The compound, having a shorter alkynyl chain, was found to be more cytotoxic to the cancer cell lines. A terminal triple bond seems to be necessary for a good activity. The cytotoxic activity of compound **3d** is similar to that of **3b** and it is lower than that observed for **3a**. This indicates that ethyl formate and 3-butynyl formate substituents at position 28 had a similar effect on the antiproliferative activity of these compounds. The structure-activity relationships indicated that the rank order of the cytotoxic activity observed in monoesters **3**, according to the nature of the formate substituent is as follows: propargyl > 3-butynyl = ethyl > 2-butynyl. The introduction of a second formate group at the C-3 position in monoesters **3** led to the complete loss of the activity (compounds **4**).

Replacement of the hydroxyl group at C-28 position in betulin (**1**) by a propynoyloxy function, *i.e*., monoester **5a**, resulted in increase of activity, especially against the cells of CCRF/CEM, with IC_50_ value of 0.02 μg/mL. It is interesting to note that compound **5a** was 545 times more cytotoxic than betulin (**1**) and 100 times more cytotoxic than cisplatin against CCRF/CEM leukemia cells. It was also observed that **5a** was more selective (SI = 15) towards CCRF/CEM than betulin (**1**). These results suggest that the propynoyloxy function is important for anti-leukemia activity. On the other hand, addition of a second propynoyl group at C-3 in monoester **5a** led to a decrease of activity except for the activity against the SW707 colon cancer cell (compound **6a**). 

**Figure 3:**
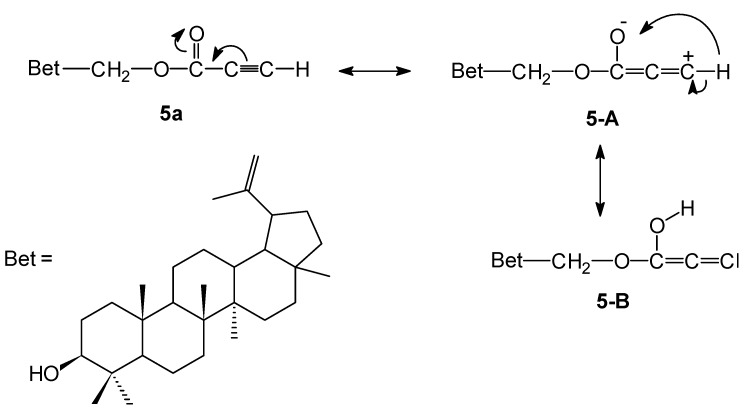
Proposed mesomeric structures of propynoyl pharmacophore of 28-*O*-propynoylbetulin (**5a**).

Though the exact reasons for this influence of propynoyl group are not quite well understood, we assume that the cytotoxic activity of compound **5a** may be a consequence of the higher electrophilicity of its triple bond. We suggest that due to the strong negative effect of the carbonyl group, the mesomeric formula describing such a delocalization of the electron density of the triple bond has a cumulene structure **5-A** and carbene structure **5-B** (Figure 3) and it can interact very easily with nucleophilic reactive groups in living systems such as amines or thiols. In order to understand the effect of the triple bond of the propynoyl pharmacophore, derivative **5c** was tested. It is interesting to note that replacement of the ethynyl group (compound **5a**) by the ethyl group (compound **5c**) resulted in a decrease of activity, especially against leukemia (CCRF/CEM and P388) cell lines. These results suggested that the triple bond directly bonded to carbonyl group of the substituent at the C-28 position seem to be essential for anticancer activity. This finding is in a good agreement with results from Csuk and co-workers showing that the 28-etynylbetulin and its carbonyl derivatives exhibited a higher cytotoxic activity compared to betulinic acid [23]. It was also reported that several acetylenic natural products possessing fungicidal properties have a conjugation of the triple bond to an adjacent carbonyl function [32]. However, the mono- **5b** and diester **6b** containing phenylproynoyl groups are inactive. The structure-activity relationships observed in compounds **5** indicated that the rank order of the cytotoxic activity, against all cancer cell lines applied, according to the nature of the acyloxy substituent, is as follows: propynoyl > propanoyl > phenylpropynoyl. It indicates that the betulins where the carbonyl group (at C-28 position) is directly bonded to triple bond of the ethynyl substituent, show a strong cytotoxic effect especially against human leukemia (CCRF/CEM) cancer cells with SI value equal to 15 and higher than betulin (**1**). 

The 28-*O*-acetylbetulin **7a** showed a higher activity than the parent compound **1**. But the introduction of the acetyl group at position C-3 in the compound **7a** significantly decreased the cytotoxicity (compound **7b**) in all the tested cell lines when compared to betulin (**1)** and **7a**. These results are consistent with the reported data for the anticancer activity *in vitro* of acetyl derivatives of betulin [33]. On the other hand, esterification of the hydroxyl group at the C-3 of 28-*O*-acetylbetulin (**7a**) with acetylenic chloroformates led to the total inactive compounds **8**. The obtained results also suggest that the free C-3 hydroxyl function group is important for the cytotoxicity of studied compounds.

## 3. Experimental

### 3.1. General Techniques

Melting points were determined in open capillary tubes on a Boetius melting point apparatus and are uncorrected. NMR spectra (600/150 MHz) were recorded on a Bruker MSL 600 spectrometer in CDCl_3_ solvents with tetramethylsilane as internal standard; chemical shifts are reported in ppm (δ) and *J* values in Hz. Multiplicity is designated as singlet (s), doublet (d), triplet (t), quartet (q), multiplet (m). Mass spectra were recorded under EI conditions on a Finnigan MAT 95 instrument, IR spectra (KBr, pellet) on a IRAffinity-1 Shimadzu spectrophotometer. Elemental C, H analyses were obtained on a Carlo Erba Model 1108 analyzer. TLC was performed on silica gel 60 254F plates (Merck, Darmstadt, Germany) using a mixture of chloroform and ethanol (20:1 or 40:1, v/v) as an eluent. Spots were visualized by spraying with solution of 5% sulfuric acid, followed by heating. Column chromatography was performed on silica gel 60, <63 μm (Merck) using a mixture of chloroform and ethanol (40:1, v/v) as an eluent. Solvents were dried and purified according to usual procedures.

### 3.2. Isolation of Betulin (**1**)

External bark (100 g) of the white birch (*Betula verrucosa*), collected in Poland, was soaked in dichloromethane (1 L) and refluxed for 8 hours. The solvent was removed under reduced pressure and the crude product was purified by column chromatography (chloroform/ethanol, 20:1, v/v) to give **1** as a white solid (19 g, 19%): m.p. 250–252 °C (lit. m.p. 252–253 °C [6]; 255–256 °C [27]); R_f_ 0.42 (chloroform/ethanol, 20:1, v/v). The ^1^H- and ^13^C-NMR spectral data of **1** were in agreement with those published in the literature [6].

### 3.3. General Procedure for the Synthesis of Monoesters **3** and Diesters **4**

To a mixture of betulin (**1**, 0.44 g, 1 mmol) and pyridine (2.5 mL) in dry benzene (6 mL) at 0–5 °C, a solution of propargyl chloroformate or 2-butyn-1-yl chloroformate or 3-butyn-1-yl chloroformate or ethyl chloroformate (3.0 mmol) in dry benzene (5 mL) was added. Stirring at this temperature was continued for 4 h. Then the reaction mixture was allowed to warm to room temperature and stirred over night. At the end of the reaction 5 mL of chloroform was added and the reaction mixture was washed with 1 N sulfuric acid, followed by water, then dried with anhydrous magnesium sulfate and concentrated under reduced pressure. The crude product was purified by column chromatography (chloroform/ethanol, 40:1, v/v) to give pure corresponding monoesters **3** and diesters **4**.

*28-O-Propargyloxycarbonylbetulin* (**3a**). Yield: 69%, m.p. 190–193 °C, R_f_ 0.49 (chloroform/ethanol, 40:1, v/v); ^1^H-NMR (CDCl_3_) δ: 0.76 (s, 3H, CH_3_), 0.82 (s, 3H, CH_3_), 0.97 (s, 3H, CH_3_), 0.98 (s, 3H, CH_3_), 1.03 (s, 3H, CH_3_), 1.68 (s, 3H, CH_3_), 1.06–2.01 (m, 25H, CH, CH_2_), 2.42 (m, 1H, H-19), 2.53 (t, *J* = 2.4 Hz, 1H, C≡C*H*), 3.18 (m, 1H, H-3), 3.95 (d, *J* = 10.8 Hz, 1H, H-28), 4.38 (d, *J* = 10.8 Hz, 1H, H-28), 4.59 (s, 1H, H-29), 4.69 (s, 1H, H-29), 4.74 (d, *J* = 2.4 Hz, 2H, OC*H*_2_). ^13^C-NMR (CDCl_3_) δ: 14.9, 15.3, 15.4, 15.9, 16.2, 18.3, 19.1, 20.8, 25.2, 26.9, 27.4, 27.9, 28.0, 29.5, 34.2, 34.3, 37.1, 37.6, 38.7, 38.8. 40.8, 42.7, 46.5, 47.6, 48.8, 50.3, 55.2, 55.4, 67.3, 75.6, 78.9, 109.9, 149.9, 155.1. IR (KBr, cm^−1^) ν: 3421, 3309, 2132, 1749, 1250, 885. EI MS (70 eV) *m/z* (rel. intensity): 524 (M^+^, 11), 411 (45), 207 (59), 203 (58), 189 (100), 135 (58); Elemental anal. (%), calcd. for C_34_H_52_O_4_: C, 77.82; H, 9.99; found: C, 77.59; H, 9.84.

*28-O-(3-Butynyloxycarbonyl)betulin* (**3b**). Yield: 64%, m.p. 86–88 °C, R_f_ 0.45 (chloroform/ethanol, 40:1, v/v); ^1^H-NMR (CDCl_3_) δ: 0.76 (s, 3H, CH_3_), 0.82 (s, 3H, CH_3_), 0.97 (s, 3H, CH_3_), 0.98 (s, 3H, CH_3_), 1.03 (s, 3H, CH_3_), 1.68 (s, 3H, CH_3_), 1.05–2.02 (m, 25H, CH, CH_2_), 2.03 (t, *J* = 2.7 Hz, 1H, C≡C*H*), 2.43 (m, 1H, H-19), 2.59 (m, 2H, *J* = 2.7 Hz, *J* = 7.2 Hz, OCH_2_C*H*_2_), 3.19 (m, 1H, H-3), 3.93 (d, *J* = 10.8 Hz, 1H, H-28), 4.25 (t, *J* = 7.2 Hz, 2H, OC*H*_2_), 4.36 (d, *J* = 10.8 Hz, 1H, H-28), 4.59 (s, 1H, H-29), 4.69 (s, 1H, H-29). ^13^C-NMR (CDCl_3_) δ: 14.7, 15.3, 15.9, 16.1, 18.2, 19.0, 20.7, 25.1, 26.9, 27.3, 27.9, 29.4, 34.1, 34.3, 37.1, 37.5, 38.6, 38.8, 40.8, 42.6, 46.5, 47.6, 48.7, 50.3, 55.2, 65.3, 66.7, 70.2, 76.7, 78.9, 79.4, 109.9, 150.0, 155.4. IR (KBr, cm^−1^) ν: 3485, 3310, 2124, 1747, 1248, 884. EI MS (70 eV) *m/z* (rel. intensity): 538 (M^+^, 10), 207 (47), 203 (52), 189 (100), 135 (57); Elemental anal. (%), calcd. for C_35_H_54_O_4_: C, 78.02; H, 10.10; found: C, 78.32; H, 9.98.

*28-O-(2-Butynyloxycarbonyl)betulin* (**3c**). Yield: 54%, m.p. 112–114 °C, R_f_ 0.48 (chloroform/ethanol, 40:1, v/v); ^1^H-NMR (CDCl_3_) δ: 0.76 (s, 3H, CH_3_), 0.82 (s, 3H, CH_3_), 0.96 (s, 3H, CH_3_), 0.97 (s, 3H, CH_3_), 1.03 (s, 3H, CH_3_), 1.68 (s, 3H, CH_3_), 1.06–2.01 (m, 25H, CH, CH_2_), 1.87 (t, *J* = 2.4 Hz, 3H, C≡CC*H*_3_), 2.42 (m, 1H, H-19), 3.19 (m, 1H, H-3), 3.94 (d, *J* = 10.8 Hz, 1H, H-28), 4.37 (d, *J* = 10.8 Hz, 1H, H-28), 4.59 (s, 1H, H-29), 4.69 (s, 1H, H-29), 4.70 (q, 2H, *J* = 2.4 Hz, OC*H*_2_). ^13^C-NMR (CDCl_3_) δ: 3.7, 14.7, 15.3, 15.9, 16.1, 18.2, 19.0, 20.7, 25.1, 26.9, 27.3, 27.9, 29.4, 29.4, 34.1, 34.3, 37.1, 37.5, 38.6, 38.8, 40.8, 42.6, 46.5, 47.6, 48.7, 50.2, 55.2, 56.1, 66.9, 72.5, 76.7, 78.9, 109.9, 150.0, 155.2. IR (KBr, cm^−1^) ν: 3462, 2942, 2248, 1748, 1248, 883. EI MS (70 eV) *m/z* (rel. intensity): 538 (M^+^, 14), 207 (48), 203 (53), 189 (100), 135 (60); Elemental anal. (%), calcd. for C_35_H_54_O_4_: C, 78.02; H, 10.10; found: C, 77.84; H, 10.22.

*28-O-Ethoxycarbonylbetulin* (**3d**). Yield: 68%, m.p. 94–96 °C, R_f_ 0.47 (chloroform/ethanol, 40:1, v/v); ^1^H-NMR (CDCl_3_) δ: 0.75 (s, 3H, CH_3_), 0.81 (s, 3H, CH_3_), 0.96 (s, 3H, CH_3_), 0.97 (s, 3H, CH_3_), 1.03 (s, 3H, CH_3_), 1.31 (t, *J* = 7.2 Hz, 3H, OCH_2_C*H*_3_), 1.67 (s, 3H, CH_3_), 1.05–2.02 (m, 25H, CH, CH_2_), 2.42 (m, 1H, H-19), 3.18 (m, 1H, H-3), 3.90 (d, *J* = 10.8 Hz, 1H, H-28), 4.20 (q, *J* = 7.2 Hz, 2H, OC*H*_2_), 4.32 (d, *J* = 10.8 Hz, 1H, H-28), 4.58 (s, 1H, H-29), 4.68 (s, 1H, H-29). ^13^C-NMR (CDCl_3_) δ: 14.2, 14.7, 15.3, 15.9, 16.0, 18.2, 19.0, 20.7, 25.1, 26.9, 27.3, 27.9, 29.5, 34.1, 34.3, 37.1, 37.5, 38.6, 38.8, 40.8, 42.6, 46.4, 47.6, 48.7, 50.3, 55.2, 63.9. 66.3, 78.9, 109.8, 150.0, 155.7. IR (KBr, cm^−1^) ν: 3486, 2942, 1744, 1249, 1013, 882. EI MS (70 eV) *m/z* (rel. intensity): 514 (M^+^, 18), 424 (40), 203 (54), 189 (100), 135 (56); Elemental anal. (%), calcd. For C_33_H_54_O_4_: C, 77.00; H, 10.57; found: C, 77.28; H, 10.43.

*3,28-O,O'-Di(propargyloxycarbonyl)betulin* (**4a**). Yield: 27%, m.p. 125–127 °C, R_f_ 0.77 (chloroform/ethanol, 40:1, v/v); ^1^H-NMR (CDCl_3_) δ: 0.84 (s, 3H, CH_3_), 0.85 (s, 3H, CH_3_), 0.91 (s, 3H, CH_3_), 0.96 (s, 3H, CH_3_), 1.02 (s, 3H, CH_3_), 1.67 (s, 3H, CH_3_), 1.07–2.02 (m, 25H, CH, CH_2_), 2.42 (m, 1H, H-19), 2.51 (t, *J* = 2.4 Hz, 1H, C≡C*H*), 2.53 (t, *J* = 2.4 Hz, 1H, C≡C*H*), 3.94 (d, *J* = 10.8 Hz, 1H, H-28), 4.33 (m, 1H, H-3), 4.37 (d, *J* = 10.8 Hz, 1H, H-28), 4.59 (s, 1H, H-29), 4.68 (s, 1H, H-29), 4.73 (d, *J* = 2.4 Hz, 2H, OC*H*_2_), 4.76 (d, *J* = 2.4, Hz, 2H, OC*H*_2_). ^13^C-NMR (CDCl_3_) δ: 15.9, 16.0, 16.1, 16.2, 16.3, 18.0, 19.0, 20.7, 23.5, 25.1, 27.0, 27.7, 27.9, 29.4, 34.0, 34.3, 37.0, 37.6, 38.0, 38.3, 40.8, 42.7, 46.5, 47.6, 48.7, 50.2, 54.9, 55.2, 55.6, 67.2, 75.6, 75.9, 77.0, 86.2, 109.9, 149.9, 153.9, 154.5. IR (KBr, cm^−1^) ν: 3305, 2941, 2133, 1730, 1442, 1253. EI MS (70 eV) *m/z* (rel. intensity): 606 ((M^+^, 7), 506 (41), 203 (52), 189 (100), 121 (42); Elemental anal. (%), calcd. for C_38_H_54_O_6_: C, 75.21; H, 8.97; found: C, 75.03; H 9.12.

*3,28-O,O'-Di(3-butynyloxycarbonyl)betulin* (**4b**). Yield: 23%, m.p. 140–142 °C, R_f_ 0.75 (chloroform/ ethanol, 40:1, v/v); ^1^H-NMR (CDCl_3_) δ: 0.84 (s, 3H, CH_3_), 0.85 (s, 3H, CH_3_), 0.91 (s, 3H, CH_3_), 0.97 (s, 3H, CH_3_), 1.03 (s, 3H, CH_3_), 1.68 (s, 3H, CH_3_), 1.05–2.02 (m, 25H, CH, CH_2_), 2.01 (t, *J* = 2.4 Hz, 1H, C≡C*H*), 2.03 (t, *J* = 2.4 Hz, 1H, C≡C*H*), 2.43 (m, 1H, H-19), 2.59 (m, 4H, 2 × OCH_2_C*H*_2_), 3.94 (d, *J* = 10.8 Hz, 1H, H-28), 4.24 (m, 4H, 2 × OC*H*_2_), 4.31 (m, 1H, H-3), 4.36 (d, *J* = 10.8 Hz, 1H, H-28), 4.59 (s, 1H, H-29), 4.69 (s, 1H, H-29). ^13^C-NMR (CDCl_3_) δ: 14.6, 15.9, 16.1, 16.3, 18.0, 19.0, 20.7, 23.5, 25.0, 26.9, 27.8, 29.4, 33.9, 34.3, 36.9, 37.5, 38.0, 38.2, 40.8, 42.6, 46.4, 47.6, 48.7, 50.1, 55.3, 64.9, 65.3, 66.7, 70.1, 70.2, 76.7, 79.4, 79.5, 79.4, 85.6, 109.9, 149.9, 154.9, 155.4. IR (KBr, cm^−1^) ν: 3312, 2951, 2128, 1745, 1249, 884. EI MS (70 eV) *m/z* (rel. intensity): 634 (M^+^, 3), 203 (40), 189 (100), 135 (48), 121 (46); Elemental anal. (%), calcd. for C_40_H_58_O_6_: C, 75.67; H, 9.21; found: C, 75.89; H, 9.14.

*3,28-O,O'-Di(2-butynyloxycarbonyl)betulin* (**4c**). Yield: 28%, m.p. 70–73 °C, R_f_ 0.78 (chloroform/ ethanol, 40:1, v/v); ^1^H-NMR (CDCl_3_) δ: 0.84 (s, 3H, CH_3_), 0.85 (s, 3H, CH_3_), 0.91 (s, 3H, CH_3_), 0.97 (s, 3H, CH_3_), 1.03 (s, 3H, CH_3_), 1.68 (s, 3H, CH_3_), 1.05–2.02 (m, 25H, CH, CH_2_), 1.86 (t, *J* = 2.4 Hz, 3H, C≡CC*H*_3_), 1.87 (t, *J* = 2.4 Hz, 3H, C≡CC*H*_3_), 2.42 (m, 1H, H-19), 3.94 (d, *J* = 10.8 Hz, 1H, H-28), 4.32 (m, 1H, H-3), 4.37 (d, *J* = 10.8 Hz, 1H, H-28), 4.59 (s, 1H, H-29), 4.69 (s, 1H, H-29), 4.71 (m, 4H, 2 × OC*H*_2_). ^13^C-NMR (CDCl_3_) δ: 5.1, 5.3, 15.9, 16.1, 16.3, 18.0, 19.0, 20.7, 23.5, 25.0, 26.9, 27.8, 29.4, 33.9, 34.2, 36.9, 37.5, 37.9, 38.2, 40.8, 42.6, 46.4, 47.6, 48.7, 50.1, 55.3, 55.8, 56.1, 56.4, 66.9, 72.5, 72.7, 83.9, 84.1, 84.2, 85.8, 109.9, 149.9, 154.7, 155.2. IR (KBr, cm^−1^) ν: 2948, 2241, 1747, 1456, 1265, 884. EI MS (70 eV) *m/z* (rel. intensity): 634 (M^+^, 6), 520 (94), 203 (42), 189 (100), 135 (41); Elemental anal. (%), calcd. for C_40_H_58_O_6_: C, 75.67; H, 9.21; found: C, 75.42; H, 9.33.

*3,28-O,O'-Di(ethoxycarbonyl)betulin* (**4d**). Yield: 24%, m.p. 84–86 °C, R_f_ 0.76 (chloroform/ethanol, 40:1, v/v); ^1^H-NMR (CDCl_3_) δ: 0.86 (s, 3H, CH_3_), 0.87 (s, 3H, CH_3_), 0.93 (s, 3H, CH_3_), 0.99 (s, 3H, CH_3_), 1.05 (s, 3H, CH_3_), 1.32 (m, 6H, 2 × OCH_2_C*H*_3_), 1.69 (s, 3H, CH_3_), 0.81–2.05 (m, 25H, CH, CH_2_), 2.44 (m, 1H, H-19), 3.92 (d, *J* = 10.8 Hz, 1H, H-28), 4.22 (m, 4H, 2 × OC*H*_2_), 4.31 (m, 1H, H-3) 4.35 (d, *J* = 10.8 Hz, 1H, H-28), 4.60 (s, 1H, H-29), 4.70 (s, 1H, H-29). ^13^C-NMR (CDCl_3_) δ: 14.2, 14.7, 16.0, 16.1, 16.4, 18.1, 19.1, 20.8, 23.6, 25.1, 27.0, 27.8, 29.5, 34.1, 34.3, 37.0, 37.6, 38.0, 38.3, 40.9, 42.7, 46.5, 47.6, 48.8, 50.2, 55.4, 63.6, 63.9, 66.4, 85.1, 109.9, 150.0, 155.2, 155.7. IR (KBr, cm^−1^) ν: 2948, 1741, 1466, 1265, 1009, 885. EI MS (70 eV) *m/z* (rel. intensity): 586 (M^+^, 4), 496 (70), 203 (42), 189 (100), 135 (41). Elemental anal. (%), calcd. for C_36_H_58_O_6_: C, 73.68; H, 9.96; found: C, 73.56; H, 9.85.

### 3.4. General Procedure for the Synthesis of Monoesters **5** and Diesters **6**

To a mixture of betulin (**1**, 0.44 g, 1 mmol) and propynoic acid (0.08 g, 1.10 mmol), phenylpropynoic acid (0.16 g, 1.10 mmol) or propanoic acid (0.08 g, 1.10 mmol) in dry dichloromethane (5 mL) under argon at −10 °C, a solution of DCC (0.23 g, 1.12 mmol) and DMAP (0.01 g, 0.08 mmol) in dry dichloromethane (1 mL) was added. Stirring at this temperature was continued for 5 h. Then the reaction mixture was allowed to warm to room temperature and stirred over night. After the reaction was complete, the reaction mixture was filtered and the solvent was removed under reduced pressure. The crude product was purified by column chromatography (chloroform/ethanol 40:1, v/v) to give pure corresponding monoesters **5** and diesters **6**.

*28-O-Propynoylbetulin* (**5a**). Yield: 60%, m.p. 133–135 °C, R_f_ 0.46 (chloroform/ethanol, 20:1, v/v); ^1^H-NMR (CDCl_3_) δ: 0.76 (s, 3H, CH_3_), 0.82 (s, 3H, CH_3_), 0.97 (s, 3H, CH_3_), 0.98 (s, 3H, CH_3_), 1.03 (s, 3H, CH_3_), 1.68 (s, 3H, CH_3_), 1.06–1.98 (m, 25H, CH, CH_2_), 2.43 (m, 1H, H-19), 2.89 (s, 1H, C≡C*H*), 3.18 (m, 1H, H-3), 3.99 (d, *J* = 10.8 Hz, 1H, H-28), 4.38 (d, *J* = 10.8 Hz, 1H, H-28), 4.59 (s, 1H, H-29), 4.69 (s, 1H, H-29). ^13^C-NMR (CDCl_3_) δ: 14.7, 15.3, 15.9, 16.0, 18.2, 19.1, 20.7, 25.1, 26.9, 27.3, 27.9, 29.4, 29.5, 34.1, 34.4, 37.1, 37.6, 38.6, 38.8, 40.8, 42.6, 46.3, 47.6, 48.7, 50.3, 55.2, 64.8, 74.6, 74.6, 78.9, 109.9, 149.8, 153.2. IR (KBr, cm^−1^) ν: 3419, 3302, 2994, 2120, 1715, 1227. EI MS (70 eV) *m/z* (rel. intensity): 494 (M^+^, 32), 207 (66), 203 (50), 189 (100), 135 (59); Elemental anal. (%), calcd. for C_33_H_50_O_3_: C, 80.11; H, 10.19; found: C, 80.39; H, 10.02.

*28-O-Phenylpropynoylbetulin* (**5b**). Yield: 70%, m.p. 204–206 °C, R_f_ 0.47 (chloroform/ethanol, 20:1, v/v); ^1^H-NMR (CDCl_3_) δ: 0.76 (s, 3H, CH_3_), 0.83 (s, 3H, CH_3_), 0.97 (s, 3H, CH_3_), 0.99 (s, 3H, CH_3_), 1.05 (s, 3H, CH_3_), 1.69 (s, 3H, CH_3_), 1.07–2.05 (m, 25H, CH, CH_2_), 2.46 (m, 1H, H-19), 3.18 (m, 1H, H-3), 4.04 (d, *J* = 10.8 Hz, 1H, H-28), 4.42 (d, *J* = 10.8 Hz, 1H, H-28), 4.60 (s, 1H, H-29), 4.70 (s, 1H, H-29), 7.36–7.60 (m, 5H, Ar-H). ^13^C-NMR (CDCl_3_) δ: 14.7, 15.3, 16.0, 16.1, 18.2, 19.1, 20.7, 25.1, 27.0, 27.3, 27.9, 29.5, 29.6, 34.1, 34.5, 37.1, 37.6, 38.6, 38.8, 40.8, 42.7, 46.4, 47.6, 48.8, 50.3, 55.2, 64.5, 78.9, 80.7, 86.2, 109.9, 119.6, 128.5, 128.5, 130.5, 132.9, 132.9, 149.9, 154.7. IR (KBr, cm^−1^) ν: 3587, 2993, 2220, 1691, 1287, 1195. EI MS (70 eV) *m/z* (rel. intensity): 570 (M^+^, 15), 189 (71), 135 (42), 129 (100), 95 (41); Elemental anal. (%), calcd. for C_39_H_54_O_3_: C, 82.06; H, 9.53; found: C, 82.31; H, 9.48.

*28-O-Propanoylbetulin* (**5c**). Yield: 86%, m.p. 160–162 °C, R_f_ 0.46 (chloroform/ethanol, 20:1, v/v); ^1^H-NMR (CDCl_3_) δ: 0.75 (s, 3H, CH_3_), 0.81 (s, 3H, CH_3_), 0.96 (s, 3H, CH_3_), 0.97 (s, 3H, CH_3_), 1.02 (s, 3H, CH_3_), 1.15 (t, *J* = 7.2 Hz, 3H, CH_2_C*H*_3_), 1.67 (s, 3H, CH_3_), 0.88–1.93 (m, 25H, CH, CH_2_), 2.34 (q, *J* = 7.2 Hz, 2H, C*H*_2_CH_3_), 2.43 (m, 1H, H-19), 3.18 (m, 1H, H-3), 3.84 (d, *J* = 10.8 Hz, 1H, H-28), 4.25 (d, *J* = 10.8 Hz, 1H, H-28), 4.58 (s, 1H, H-29), 4.68 (s, 1H, H-29). ^13^C-NMR (CDCl_3_) δ: 9.2, 14.7, 15.3, 15.9, 16.0, 18.2, 19.1, 20.7, 25.1, 27.0, 27.3, 27.7, 27.9, 29.5, 29.7, 34.1, 34.5, 37.1, 37.5, 38.6, 38.8, 40.8, 42.6, 46.3, 47.6, 48.7, 50.3, 55.2, 62.5, 78.9, 109.8, 150.1, 174.9. IR (KBr, cm^−1^) ν: 3357, 2940, 1735, 1276, 1186, 882. EI MS (70 eV) *m/z* (rel. intensity): 498 (M^+^, 17), 207 (52), 203 (54), 189 (100), 135 (58); Elemental anal. (%), calcd. for C_33_H_54_O_3_: C, 79.46; H, 10.91; found: C, 79.34; H, 10.82.

*3,28-O,O'-Dipropynoylbetulin* (**6a**). Yield: 12%, m.p. 102–104 °C, R_f_ 0.73 (chloroform/ethanol, 20:1, v/v); ^1^H-NMR (CDCl_3_) δ: 0.86 (s, 3H, CH_3_), 0.87 (s, 3H, CH_3_), 0.89 (s, 3H, CH_3_), 0.98 (s, 3H, CH_3_), 1.03 (s, 3H, CH_3_), 1.68 (s, 3H, CH_3_), 1.07–2.01 (m, 25H, CH, CH_2_), 2.43 (m, 1H, H-19), 2.85 (s, 1H, C≡C*H*), 2.89 (s, 1H, C≡C*H*), 3.99 (d, *J* = 10.8 Hz, 1H, H-28), 4.39 (d, *J* = 10.8 Hz, 1H, H-28), 4.59 (m, 1H, H-3), 4.60 (s, 1H, H-29), 4.69 (s, 1H, H-29). ^13^C-NMR (CDCl_3_) δ: 14.7, 15.9, 16.1, 16.4, 18.1, 19.1, 20.7, 23.4, 25.1, 26.9, 27.8, 29.4, 29.5, 34.0, 34.4, 37.0, 37.6, 37.9, 38.3, 40.8, 42.6, 46.3, 47.6, 48.7, 50.2, 55.3, 64.8, 74.1, 74.6, 74.7, 75.1, 83.6, 110.0, 149.8, 152.7, 153.2. IR (KBr, cm^−1^) ν: 3301, 2945, 2119, 1714, 1234, 886. EI MS (70 eV) *m/z* (rel. intensity): 546 (M^+^, 9), 195 (46), 189 (53), 113 (41), 55 (100); Elemental anal. (%), calcd. for C_36_H_50_O_4_: C, 79.08; H, 9.22; found: C, 79.16; H, 9.28.

*3,28-O,O'-Di(phenylpropynoyl)betulin* (**6b**). Yield: 27%, m.p. 91–92 °C, R_f_ 0.79 (chloroform/ethanol, 20:1, v/v); ^1^H-NMR (CDCl_3_) δ: 0.88 (s, 3H, CH_3_), 0.92 (s, 3H, CH_3_), 0.93 (s, 3H, CH_3_), 0.99 (s, 3H, CH_3_), 1.06 (s, 3H, CH_3_), 1.70 (s, 3H, CH_3_), 1.06–2.05 (m, 25H, CH, CH_2_), 2.46 (m, 1H, H-19), 4.04 (d, *J* = 10.8 Hz, 1H, H-28), 4.42 (d, *J* = 10.8 Hz, 1H, H-28), 4.61 (s, 1H, H-29), 4.66 (m, 1H, H-3), 4.71 (s, 1H, H-29), 7.36–7.60 (m, 10H, 2 × Ar-H). ^13^C-NMR (CDCl_3_) δ: 14.7, 16.0, 16.1, 16.5, 18.1, 19.1, 20.8, 23.6, 25.1, 25.8, 25.9, 27.0, 27.9, 29.4, 29.5, 29.6, 34.1, 34.5, 37.0, 37.6, 38.0, 38.4, 40.9, 42.7, 46.4, 47.6, 48.8, 50.2, 55.4, 64.5, 80.7, 81.0, 83.2, 85.7, 86.3, 109.9, 119.6, 119.8, 128.4, 128.5, 130.4, 130.5, 132.9, 132.9, 149.9, 154.2, 154.7. IR (KBr, cm^−1^) ν: 2945, 2223, 1706, 1490, 1285, 1187. EI MS (70 eV) *m/z* (rel. intensity): 698 (M^+^, 6), 552 (30), 189 (33), 135 (15), 129 (100); Elemental anal. (%), calcd. for C_48_H_58_O_4_: C, 82.48; H, 8.36; found: C, 82.35; H, 8.27.

*3,28-O,O'-Dipropanoylbetulin* (**6c**). Yield: 11%, m.p. 142–144 °C, R_f_ 0.71 (chloroform/ethanol, 20:1, v/v); ^1^H-NMR (CDCl_3_) δ: 0.83 (s, 3H, CH_3_), 0.83 (s, 3H, CH_3_), 0.84 (s, 3H, CH_3_), 0.96 (s, 3H, CH_3_), 1.02 (s, 3H, CH_3_), 1.15 (m, 6H, 2 × CH_2_C*H*_3_), 1.68 (s, 3H, CH_3_), 1.06–1.96 (m, 25H, CH, CH_2_), 2.34 (m, 4H, 2 × C*H*_2_CH_3_), 2.43 (m, 1H, H-19), 3.83 (d, *J* = 10.8 Hz, 1H, H-28), 4.26 (d, *J* = 10.8 Hz, 1H, H-28), 4.65 (m, 1H, H-3), 4.58 (s, 1H, H-29), 4.68 (s, 1H, H-29). ^13^C-NMR (CDCl_3_) δ: 9.2, 9.3, 14.7, 15.9, 16.1, 16.5, 18.1, 19.1, 20.7, 23.6, 25.1, 27.0, 27.7, 27.9, 28.0, 29.5, 29.7, 34.0, 34.5, 37.0, 37.4, 37.8, 38.3, 40.8, 42.6, 46.3, 47.6, 48.7, 50.2, 55.3, 62.5, 80.5, 109.8, 150.1, 174.3, 174.9. IR (KBr, cm^−1^) ν: 2960, 1736, 1461, 1261, 1185, 891. EI MS (70 eV) *m/z* (rel. intensity): 554 (M^+^, 7), 480 (100), 203 (45), 189 (98), 135 (37); Elemental anal. (%), calcd. for C_36_H_58_O_4_: C, 77.93; H, 10.54; found: C, 77.85; H, 10.62.

### 3.5. Synthesis of 28-O-Acetylbetulin (**7a**) and 3,28-O,O'-Diacetylbetulin (**7b**)

To a mixture of betulin (**1**, 1.0 g, 2.26 mmol), DMAP (0.01 g, 0.07 mmol) and pyridine (1.5 mL) in dry dichloromethane (9 mL) was added dropwise acetic anhydride (0.24 g, 2.26 mmol) at 0 °C, and the reaction mixture was stirred at room temperature. After being stirred for 18 h, the mixture was washed with 10% aqueous hydrochloric acid (30 mL), water (15 mL), saturated aqueous NaHCO_3_ solution and then brine (20 mL). After separation of phases, the organic layer was dried with anhydrous magnesium sulfate and concentrated under reduced pressure. The crude product was purified by column chromatography (chloroform/ethanol, 40:1, v/v) to give 28-*O*-acetylbetulin (**7a**, 0.77 g, 71%): m.p. 214–215 °C (lit. m.p. 210–212 °C [28]; 217–219 °C [34]); R_f_ 0.44 (chloroform/ethanol, 20:1, v/v) and 3,28-*O,O*'-diacetylbetulin (**7b**, 0.21 g, 18%): m.p. 220–222 °C (lit. m.p. 221–223 °C [34]). ^1^H and ^13^C-NMR spectral data of **7a** and **7b** were in agreement with those published in the literature [28,34].

### 3.6. Synthesis of 28-O-acetyl-3-O'-alkynyloxycarbonylbetulins (**8**)

To a mixture of 28-*O*-acetylbetulin (**7a**, 0.48 g, 1 mmol) and pyridine (2.5 mL) in dry benzene (6 mL) at 0–5 °C, a solution of propargyl chloroformate or 2-butyn-1-yl chloroformate or 3-butyn-1-yl chloroformate or ethyl chloroformate (2.0 mmol) in dry benzene (5 mL) was added. Stirring at this temperature was continued for 4 h. Then the reaction mixture was allowed to warm to room temperature and stirred over night. At the end of the reaction 5 mL of chloroform was added and the reaction mixture was washed with 1 N sulfuric acid, followed by water, then dried with anhydrous magnesium sulfate and concentrated under reduced pressure. The crude product was purified by column chromatography using chloroform/ethanol (40:1, v/v) to give pure corresponding esters **8**.

*28-O-Acetyl-3-O'-propargyloxycarbonylbetulin* (**8a**). Yield: 89%, m.p. 188–189 °C, R_f_ 0.73 (chloroform/ ethanol, 20:1, v/v); ^1^H-NMR (CDCl_3_) δ: 0.84 (s, 3H, CH_3_), 0.85 (s, 3H, CH_3_), 0.92 (s, 3H, CH_3_), 0.97 (s, 3H, CH_3_), 1.03 (s, 3H, CH_3_), 1.68 (s, 3H, CH_3_), 1.06–1.99 (m, 25H, CH, CH_2_), 2.07 (s, 3H, COC*H*_3_), 2.44 (m, 1H, H-19), 2.51 (t, 1H, *J* = 2.4 Hz, C≡C*H*), 3.85 (d, *J* = 10.8 Hz, 1H, H-28), 4.24 (d, *J* = 10.8 Hz, 1H, H-28), 4.33 (m, 1H, H-3), 4.58 (s, 1H, H-29), 4.68 (s, 1H, H-29), 4.71 (d, 2H, *J* = 2.4 Hz, OC*H*_2_). ^13^C-NMR (CDCl_3_) δ: 14.6, 16.0, 16.1, 18.1, 19.1, 20.8, 21.0, 23.5, 25.1, 27.0, 27.7, 27.8, 29.5, 29.7, 34.1, 34.5, 37.0, 37.5, 38.0, 40.8, 42.6, 46.3, 47.7, 48.7, 50.2, 54.9, 55.6, 62.7, 75.4, 75.9, 77.2, 86.2, 109.8, 150.1, 154.5, 171.6. IR (KBr, cm^−1^) ν: 3298, 2997, 2140, 1735, 1243, 889. EI MS (70 eV) *m/z* (rel. intensity): 566 (M^+^, 7), 466 (76), 203 (46), 189 (100), 135 (40); Elemental anal. (%), calcd. for C_36_H_54_O_5_: C, 76.28; H, 9.60; found: C, 76.49; H, 9.52.

*28-O-Acetyl-3-O'-(3-butynyloxycarbonyl)betulin* (**8b**). Yield: 63%, m.p. 118–121 °C, R_f_ 0.72 (chloroform/ethanol, 20:1, v/v); ^1^H-NMR (CDCl_3_) δ: 0.83 (s, 3H, CH_3_), 0.84 (s, 3H, CH_3_), 0.90 (s, 3H, CH_3_), 0.96 (s, 3H, CH_3_), 1.02 (s, 3H, CH_3_), 1.67 (s, 3H, CH_3_), 1.06–1.99 (m, 25H, CH, CH_2_), 2.00 (t, *J* = 2.7 Hz, 1H, C≡C*H*), 2.06 (s, 3H, COC*H*_3_), 2.44 (m, 1H, H-19), 2.57 (m, *J* = 2.7 Hz, *J* = 7.2 Hz 2H, OCH_2_C*H*_2_), 3.84 (d, *J* = 10.8 Hz, 1H, H-28), 4.21 (t, *J* = 7.2 Hz, 2H, OC*H*_2_), 4.25 (d, *J* = 10.8 Hz, 1H, H-28), 4.32 (m, 1H, H-3), 4.58 (s, 1H, H-29), 4.68 (s, 1H, H-29). ^13^C-NMR (CDCl_3_) δ: 14.7, 15.9, 16.1, 16.3, 18.0, 19.0, 20.7, 21.0, 23.5, 25.0, 26.9, 27.8, 29.5, 29.6, 34.0, 34.5, 36.9, 37.4, 38.0, 38.2, 40.8, 42.6, 46.2, 47.6, 48.7, 50.2, 55.3, 62.7, 65.0, 65.5, 70.1, 79.4, 85.6, 109.8, 150.1, 154.9, 171.6. IR (KBr, cm^−1^) ν: 3284, 2944, 2212, 1741, 1249, 889. EI MS (70 eV) *m/z* (rel. intensity): 580 (M^+^, 7), 466 (66), 203 (42), 189 (100), 135 (46); Elemental anal. (%), calcd. for C_37_H_56_O_5_: C, 76.51; H, 9.72; found: C, 76.79; H, 9.59.

*28-O-Acetyl-3-O'-(2-butynyloxycarbonyl)betulin* (**8c**). Yield: 67%, m.p. 126–129 °C, R_f_ 0.75 (chloroform/ethanol, 20:1, v/v); ^1^H-NMR (CDCl_3_) δ: 0.84 (s, 3H, CH_3_), 0.85 (s, 3H, CH_3_), 0.91 (s, 3H, CH_3_), 0.97 (s, 3H, CH_3_), 1.03 (s, 3H, CH_3_), 1.68 (s, 3H, CH_3_), 1.06–1.99 (m, 25H, CH, CH_2_), 1.86 (t, *J* = 2.4 Hz, 3H, C≡CC*H*_3_), 2.07 (s, 3H, COC*H*_3_), 2.44 (m, 1H, H-19), 3.84 (d, *J* = 10.8 Hz, 1H, H-28), 4.24 (d, *J* = 10.8 Hz, 1H, H-28), 4.32 (m, 1H, H-3), 4.59 (s, 1H, H-29), 4.68 (s, 1H, H-29), 4.71 (q, *J* = 2.4 Hz, 2H, OC*H*_2_). ^13^C-NMR (CDCl_3_) δ: 3.6, 14.7, 16.1, 16.3, 18.0, 19.0, 20.7, 21.0, 23.5, 25.0, 26.9, 27.8, 29.5, 29.6, 34.0, 34.5, 36.9, 37.4, 38.0, 38.2, 40.8, 42.6, 46.2, 47.6, 48.7, 50.2, 55.3, 56.4, 62.7, 72.7, 83.8, 84.2, 85.9, 109.8, 150.1, 154.7, 171.6. IR (KBr, cm^−1^) ν: 2956, 2233, 1742, 1457, 1256, 920. EI MS (70 eV) *m/z* (rel. intensity): 580 (M^+^, 3), 466 (64), 203 (37), 189 (100), 187 (47), 135 (40); Elemental anal. (%), calcd. for C_37_H_56_O_5_: C, 76.51; H, 9.72; found: C, 76.63; H, 9.87.

*28-O-Acetyl-3-O'-(ethoxycarbonyl)betulin* (**8d**). Yield: 84%, m.p. 200–202 °C, R_f_ 0.73 (chloroform/ ethanol, 20:1, v/v); ^1^H-NMR (CDCl_3_) δ: 0.84 (s, 3H, CH_3_), 0.85 (s, 3H, CH_3_), 0.91 (s, 3H, CH_3_), 0.97 (s, 3H, CH_3_), 1.03 (s, 3H, CH_3_), 1.30 (t, *J* = 7.2 Hz, 3H, CH_2_C*H*_3_), 1.68 (s, 3H, CH_3_), 1.05–2.00 (m, 25H, CH, CH_2_), 2.07 (s, 3H, COC*H*_3_), 2.44 (m, 1H, H-19), 3.85 (d, *J* = 10.8 Hz, 1H, H-28), 4.18 (q, *J* = 7.2 Hz, 2H, OC*H*_2_), 4.24 (d, *J* = 10.8 Hz, 1H, H-28), 4.30 (m, 1H, H-3), 4.59 (s, 1H, H-29), 4.69 (s, 1H, H-29). ^13^C-NMR (CDCl_3_) δ: 14.3, 14.7, 16.0, 16.1, 16.4, 18.1, 19.1, 20.8, 21.0, 23.6, 25.1, 27.0, 27.8, 29.6, 34.1, 34.5, 37.0, 37.5, 38.0, 38.3, 40.9, 42.7, 46.3, 47.7, 48.7, 50.2, 55.4, 62.8, 63.6, 85.1, 109.8, 150.1, 155.2, 171.5. IR (KBr, cm^−1^) ν: 2941, 1741, 1446, 1253, 1012, 890. EI MS (70 eV) *m/z* (rel. intensity): 556 (M^+^, 6), 466 (73), 203 (38), 189 (100), 135 (41); Elemental anal. (%), calcd. for C_35_H_56_O_5_: C, 75.50; H, 10.14; found: C, 75.68; H, 10.04.

### 3.7. Crystal Structure Determination

X-ray diffraction measurement was performed at 100(1) K. The colorless single crystal was preselected under polarization microscope. The crystal was mounted on a glass capillary and cooled down by a cold, dry nitrogen gas stream (Oxford Cryosystem Equipment). The data were collected using SuperNova kappa diffractometer with Atlas CCD detector and CuKα radiation. Accurate cell parameters were determined and refined with CrysAlis Pro program [35]. Also for the integration of the collected data program CrysAlis Pro was used. The structure was solved using direct methods with SHELXS97 program and then refined using SHELXL-97 program [36]. Nonhydrogen atoms were refined with anisotropic displacement parameters. The hydrogen atoms were fixed geometrically at calculated distances and allowed to ride on the parent carbon or oxygen atoms.

CCDC-873113 contains the supplementary crystallographic data for this paper. These data can be obtained free of charge at http://www.ccdc.cam.ac.uk/conts/retrieving.html or from the Cambridge Crystallographic Data Centre, 12 Union Road, Cambridge CB2 1EZ, UK; Fax: +44-1223-336033; E-Mail: deposit@ccdc.cam.ac.uk.

### 3.8. Antiproliferative Assay *in Vitro*

#### 3.8.1. Cells

The following established *in vitro* cell lines were applied: SW707 (human colorectal adenocarcinoma), CCRF/CEM (human leukemia), T47D (human breast cancer), P388 (mouse leukemia) and Balb3T3 (normal mouse fibroblasts). All lines were obtained from the American Type Culture Collection (Rockville, MD, USA) and maintained at the Cell Culture Collection of the Institute of Immunology and Experimental Therapy (Wroclaw, Poland). 

Twenty-four hours before addition of the tested agents, the cells were plated in 96-well plates (Sarstedt, Numbrecht, Germany) at a density of 10^4^ cells per well in 100 μL of culture medium. The cells were cultured in the opti-MEM medium supplemented with 2 mM glutamine (Gibco, Grand Island, NY, USA), streptomycin (50 μg/mL), penicillin (50 U/mL) (both antibiotics from Polfa, Tarchomin, Poland) and 5% fetal calf serum (Gibco). The cell cultures were maintained at 37 °C in humid atmosphere saturated with 5% CO_2_.

#### 3.8.2. Compounds

The compounds were dissolved in DMSO and culture medium (1:9) to the concentration of 1 mg/mL, and subsequently diluted in culture medium to reach the required concentrations (ranging from 1 to 100 mg/mL). 

### 3.9. Antiproliferative Assay

The *in vitro* cytotoxic effect of all agents was examined using the SRB assay for adherent cells and MTT assay for leukemia cells as described by Wietrzyk *et al.* [37]. Each compound in given concentration was tested in triplicates in each experiment, which was repeated 3–5 times. The results of cytotoxic activity *in vitro* were expressed as an IC_50_ – the concentration of compound (in μg/mL) that inhibits proliferation rate of the tumor cells by 50% as compared to the control untreated cells.

## 4. Conclusions

New acetylenic derivatives of betulin containing one or two acetylenic formate or propynoyl functions at the C-3 and/or C-28 positions were synthesized. The structures of the title compounds were confirmed by ^1^H, ^13^C-NMR, IR, MS, and elemental analyses. Furthermore, the structure of 28-*O*-propynoylbetulin (**5a**) was also determined by X-ray crystal analysis. The presented studies demonstrated that a simple modification of the parent structure of betulin (**1**) can produce new potentially interesting anti-cancer agents. Among all studied compounds, 28-*O*-propynoylbetulin (**5a**) exhibited the strongest cytotoxic activity and it was over 500 times more cytotoxic than betulin (**1**) and 100 times more cytotoxic than cisplatin against CCRF/CEM cancer cells with promising selectivity index (SI = 15) under *in vitro* conditions. It was found that the betulins which possess a carbonyl group at C-28 position directly bonded to the triple bond of an ethynyl substituent, showed strong cytotoxic effects against human leukemia (CCRF/CEM) and murine leukemia (P388) cancer cells. It was also found that the free C-3 hydroxyl function is a structural requirement for the cytotoxicity of the studied compounds. The compound **5a** has been selected for further studies. Noteworthy feature of the obtained results was the observation that leukemia (CCRF/CEM and P388) cells appear to be more sensitive to the cytotoxic effects of the compounds **3** and **5** than the other cancer cells lines applied.

## References

[1] Lowitz J.T. (1788). Űber eine neue, fast benzoeartige substanz der briken. Crell’s Chem. Ann..

[2] Tolstikov G.A., Flekhter O.B., Shultz E.E., Baltina L.A., Tolstikov A.G. (2005). Betulin and its derivatives. Chemistry and biological activity. Chem. Sustain. Develop..

[3] Alakurtti S., Mäkelä T., Koskimies S., Yli-Kauhaluoma J. (2006). Pharmacological properties of the ubiquitous natural product betulin. Eur. J. Pharm. Sci..

[4] Tolstikova T.G., Sorokina I.V., Tolstikov G.A., Tolstikov A.G., Flekhter O.B. (2006). Biological activity and pharmacological prospects of lupane terpenoids: II. Semisynthetic lupane derivatives. Russ. J. Bioorg. Chem..

[5] Drebushchak T.N., Mikhailenko M.A., Brezgunova M.E., Shakhtshneider T.P., Kuznetsova S.A. (2010). Crystal structure of betulin ethanol solvate. J. Struct. Chem..

[6] Boryczka S., Michalik E., Jastrzębska M., Kusz J., Zubko M., Bębenek E. (2012). X-ray crystal structure of betulin-DMSO solvate. J. Chem. Crystallogr..

[7] Mukherjee R., Kumar V., Srivastava S.K., Agarwal S.K., Burman A.C. (2006). Betulinic acid derivatives as anticancer agents: Structure activity relationship. Anticancer Agents Med. Chem..

[8] Pisha E., Chai H., Lee I.S., Chagwedera T.E., Farnsworth N.R., Cordell G.A., Beecher C.W.W., Fong H.H.S., Kinghorn A.D., Brown D.M. (1995). Discovery of betulinic acid as a selective inhibitor of human melanoma that functions by induction of apoptosis. Nat. Med..

[9] Eiznhamer D.A., Xu Z.-Q. (2004). Betulinic acid: A promising anticancer candidate. Idrugs.

[10] Mullauer F.B., Kessler J.H., Medema J.P. (2010). Betulinic acid, a natural compound with potent anticancer effects. Anticancer Drugs.

[11] Rzeski W., Stepulak A., Szymański M., Juszczak M., Grabarska A., Sifringer M., Kaczor J., Kandefer-Szerszeń M. (2009). Betulin elicits anti-cancer effects in tumour primary cultures and cell lines *in vitro*. Basic Clin. Pharmacol. Toxicol..

[12] Dehelean C.A., Feflea S., Molnar J., Zupko I., Soica C. (2012). Betulin as an antitumor agent tested *in vitro* on A431, HeLa and MCF7, and as an angiogenic inhibitor *in vivo* in the CAM assay. Nat. Prod. Commun..

[13] Alakurtti S., Heiska T., Kiriazis A., Sacerdoti-Sierra N., Jaffe C.L., Yli-Kauhaluoma J. (2010). Synthesis and anti-leishmanial activity of heterocyclic betulin derivatives. Bioorg. Med. Chem..

[14] Urban M., Vlk M., Dzubak P., Hajduch M., Sarek J. (2012). Cytotoxic heterocyclic triterpenoids derived from betulin and betulinic acid. Bioorg. Med. Chem..

[15] Pohjala L., Alakurtti S., Ahola T., Yli-Kauhaluoma J., Tammela P. (2009). Betulin-derived compounds as inhibitors of Alphavirus replication. J. Nat. Prod..

[16] Kommera H., Kaluderović G.N., Dittrich S., Kalbitz J., Dräger B., Mueller T., Paschke R. (2010). Carbamate derivatives of betulinic acid and betulin with selective cytotoxic activity. Bioorg. Med. Chem. Lett..

[17] Ben-Zvi Z., Danon A., Patai S. (1994). Supplement C2: The Chemistry of Triple Bonded Functional Groups. The Chemistry of Functional Groups.

[18] Csuk R., Barthel A., Schwarz S., Kommera H., Paschke R. (2010). Synthesis and biological evaluation of antitumor-active γ-butyrolactone substituted betulin derivatives. Bioorg. Med. Chem..

[19] Kazakova O.B., Giniyatullina G.V., Yamansarov E.Y., Tolstikov G.A. (2010). Betulin and ursolic acid synthetic derivatives as inhibitors of *Papilloma virus*. Bioorg. Med. Chem. Lett..

[20] Csuk R., Sczepek R., Siewert B., Nitsche C. (2013). Cytotoxic betulin-derived hydroxypropargylamines trigger apoptosis. Bioorg. Med. Chem..

[21] Csuk R., Barthel A., Kluge R., Ströhl D. (2010). Synthesis, cytotoxicity and liposome preparation of 28-acetylenic betulin derivatives. Bioorg. Med. Chem..

[22] Kazakova O.B., Yamansarov E.Yu., Spirikhin L.V., Yunusov M.S., Baikova I.P., Kukovinets O.S., Musin R.Z. (2011). Effective synthesis and transformations of alkyne betulin derivatives. Russ. J. Bioorg. Chem..

[23] Csuk R., Barthel A., Sczepek R., Siewert B., Schwarz S. (2011). Synthesis, encapsulation and antitumor activity of new betulin derivatives. Arch. Pharm. Chem. Life Sci..

[24] Bori I.D., Hung H.-Y., Qian K., Chen C.-H., Morris-Natschke S.L., Lee H.-K. (2012). Anti-AIDS agents 88. Anti-HIV conjugates of betulin and betulinic acid with AZT prepared via click chemistry. Tetrahedron Lett..

[25] Mól W., Matyja M., Filip B., Wietrzyk J., Boryczka S. (2008). Synthesis and antiproliferative activity *in vitro* of novel (2-butynyl)thioquinolines. Bioorg. Med. Chem..

[26] Boryczka S., Mól W., Milczarek M., Wietrzyk J., Bębenek E. (2011). Synthesis and *in vitro* antiproliferative activity of novel (4-chloro- and 4-acyloxy-2-butynyl)thioquinolines. Med. Chem. Res..

[27] Lugemwa F.L., Huang F.-Y., Bentley M.D., Mendel M.J., Alford A.R. (1990). A *Heliothis zea* antifeedant from the abundant birchbark triterpene betulin. J. Agric. Food Chem..

[28] Deng Y., Snyder J.K. (2002). Preparation of a 24-nor-1,4-dien-3-one triterpene derivative from betulin: A new route to 24-nortriterpene analogues. J. Org. Chem..

[29] Mohammed I.E., Choudhary M.I., Ali S., Anjum S., Atta-ur-Rahman. (2006). 20(29)-Lupene-3β,28β-diacetate. Acta Crystallogr..

[30] Boryczka S., Bębenek E., Jastrzębska M., Kusz J., Zubko M. (2012). Crystal structure of betulinic acid-DMSO solvate. Z. Kristallogr..

[31] Geran R.I., Greenberg N.H., Macdonald M.M., Schumacher A.M., Abott B.J. (1972). Cell culture technical procedures. Cancer Chemother. Rep..

[32] Lamberth C. (2008). Alkyne chemistry in crop protection. Bioorg. Med. Chem..

[33] Gauthier C., Legault J., Lebrun M., Dufour P., Pichette A. (2006). Glycosidation of lupine-type triterpenoids as potent *in vitro* cytotoxic agents. Bioorg. Med. Chem..

[34] Bodrikov I.V., Borisova N.V., Chiyanov A.A., Kurskii Y.A., Fukin G.K. (2013). Vinylic substitution in the reaction of betulin diacetate with *tert*-butyl hypochlorite. Russ. J. Org. Chem..

[35] (2008). Oxford Diffraction, CrysAllis CCD and CrysAllis RED.

[36] Sheldrick G.M. (2008). A short history of SHELX. Acta Crystallogr..

[37] Wietrzyk J., Chodynski M., Fitak H., Wojdat E., Kutner A., Opolski A. (2007). Antitumor properties of diastereomeric and geometric analogs of vitamin D3. Anticancer Drugs.

